# CVD graphene/Ge interface: morphological and electronic characterization of ripples

**DOI:** 10.1038/s41598-019-48998-1

**Published:** 2019-08-29

**Authors:** Cesar D. Mendoza, Neileth S. Figueroa, Marcelo E. H. Maia da Costa, Fernando L. Freire

**Affiliations:** Departamento de Física, Pontifícia Universidade Católica do Rio de Janeiro, 22451-900 Rio de Janeiro, RJ Brazil

**Keywords:** Materials science, Condensed-matter physics

## Abstract

Graphene grown directly on germanium is a possible route for the integration of graphene into nanoelectronic devices as well as it is of great interest for materials science. The morphology of the interface between graphene and germanium influences the electronic properties and has not already been completely elucidated at atomic scale. In this work, we investigated the morphology of the single-layer graphene grown on Ge substrates with different crystallographic orientations. We determined the presence of sinusoidal ripples with a single propagation direction, zig-zag, and could arise due to compressive biaxial strain at the interface generated as a result of the opposite polarity of the thermal expansion coefficient of graphene and germanium. Local density of states measurements on the ripples showed a linear dispersion relation with the Dirac point slightly shifted with respect to the Fermi energy indicating that these out-of-plane deformations were n-doped, while the graphene regions between the highs were undoped.

## Introduction

Since the pioneering work of the Manchester’s group^1^ when graphene was isolated for the first time by using a simple mechanical exfoliation method from a highly oriented pyrolytic graphite (HOPG) sample, the graphene has shown significant potential to play an important role in nanoelectronic devices due to its outstanding properties. These properties include high carrier mobility, transparency, and thickness of only one-atom^2^. They are important for several applications: data communications^3^, high-performance light emitting diodes (LEDs)^4^, low-power photonics^5^ and ultrafast- and broad-band photo-detection from ultraviolet to the terahertz range^6^. However, all of these properties were obtained by using exfoliated samples, a process that cannot be scaled for large scale device production. For this purpose, graphene must be defectless and produced over a large area at low cost^7^.

Today, chemical vapour deposition (CVD) is the main growth technique used and is the biggest bet for achieving this goal^8^. There are many reports about the synthesis of large area single-layer graphene samples. They represent the state of the art of the synthesis by CVD processes with the production of highly crystallographic oriented single-layer graphene with sample area as large as one square meter with good electronic properties^9^, such growth techniques apparently overcome the challenges of large area graphene production. However, this method is still based on the use of conductive substrates which turn it non-viable to be used for the fabrication of electronic devices. In this way, to obtain graphene by CVD, the film must be transferred to a dielectric or semiconducting substrate. The most commonly used technique is the transfer of graphene by applying a sacrificial layer of polymer over the sample and removing the copper foil using a suitable acid solution^10^. The transfer process usually leaves some polymeric residues that are responsible for the deterioration of graphene’s transport properties^11^. Also, since it is a delicate procedure it can generate defects in the transferred layer. Besides, it was demonstrated that metallic contamination levels on the graphene layer are much higher than the accepted in the fabrication of electronic devices^12^. One alternative route is the direct synthesis of single-layer graphene on dielectric or semiconducting substrates^13,14^.

Germanium is an intrinsic semiconductor with higher carrier mobility than silicon, and its integration with graphene sheets is of high interest for both fundamental materials science and electronic device applications^15^. The growth of single-layer graphene on Ge(100) substrate was successfully achieved by both atmospheric- and low-pressure CVD (APCVD and LPCVD, respectively)^16–24^. Although this orientation (100) is really interesting for implementation into Si technology, it undergoes reconstructions into the [107] facet in both the graphene growth and/or after post-growth annealing^18,22,23^, which would limit its use in possible applications. Nevertheless, there are several works about other atomically flat orientations such as (110) and (111) that arise as models to future applications as these surface orientations are expected to facilitate the high electron and hole mobility for n- and p- field effect transistors (FETs), respectively^17,18,25^. In the last few years, the study of the graphene/Ge interface was focused on the surface reconstruction of germanium^18,26^, the interaction between graphene and germanium substrate^27^, the effect of doping of the substrate on graphene growth^28^, and graphene as a protective layer of germanium substrate^29^. However, a more complete investigation that also considers the role of crystallographic orientation and the morphology of graphene is still needed.

One important aspect is the morphological and electronic characterizations at nanoscale of the ripples formed in the graphene grown by CVD on Ge substrates, since they can have a significant influence on the electronic and magnetic properties of the graphene film^30–32^. Ripples in graphene films have been observed and studied on both freestanding and supported single-layer graphene^33–35^. These results revealed that the interaction between graphene and the substrate is an important factor to be taken into consideration for the formation of those surface corrugations. In particular, the opposite polarity of the thermal expansion coefficient - negative for graphene and positive for metals – was suggested to be responsible for the wrinkles formation by CVD^36^. Epitaxial graphene grown on silicon carbide (SiC) shows the presence of ridges at the surface due to the relaxation of compressive stress generated during the cooling step of the synthesis because of the negative thermal expansion coefficient of graphene^37^.

In this work, we have studied the morphology and electronic structure of graphene films on Ge substrates. In order to do that, we have synthesized single-layer graphene by CVD using methane as precursor on germanium (undoped-Ge(110) and p-type doped with Ga Ge(100)) substrates. The films were characterized by Raman spectroscopy and mapping, while the surface topology was investigated in atomic-scale by scanning tunneling microscopy (STM) in ultra-high vacuum at room temperature conditions as well as by atomic force microscopy (AFM). The local electronic changes were determined by scanning tunneling spectroscopy (STS). Our Raman results revealed that the graphene layer was submitted to a biaxial compressive strain. The morphology of the graphene films was characterized by the presence of sinusoidal ripples with a single propagation direction (zig-zag).

## Results and Discussion

To study the morphology and electronic structure of graphene films, two sets of samples were synthesized by CVD. The first one was obtained on Ge(110) surface and the second one was prepared on Ge(100) surface as described elsewhere^22^. More information about preparation and growth of the samples such as temperature, environment, CH_4_:H_2_ flow ratios and time are in the method section and Supplementary Information, Fig. S1.

**Figure 1:**
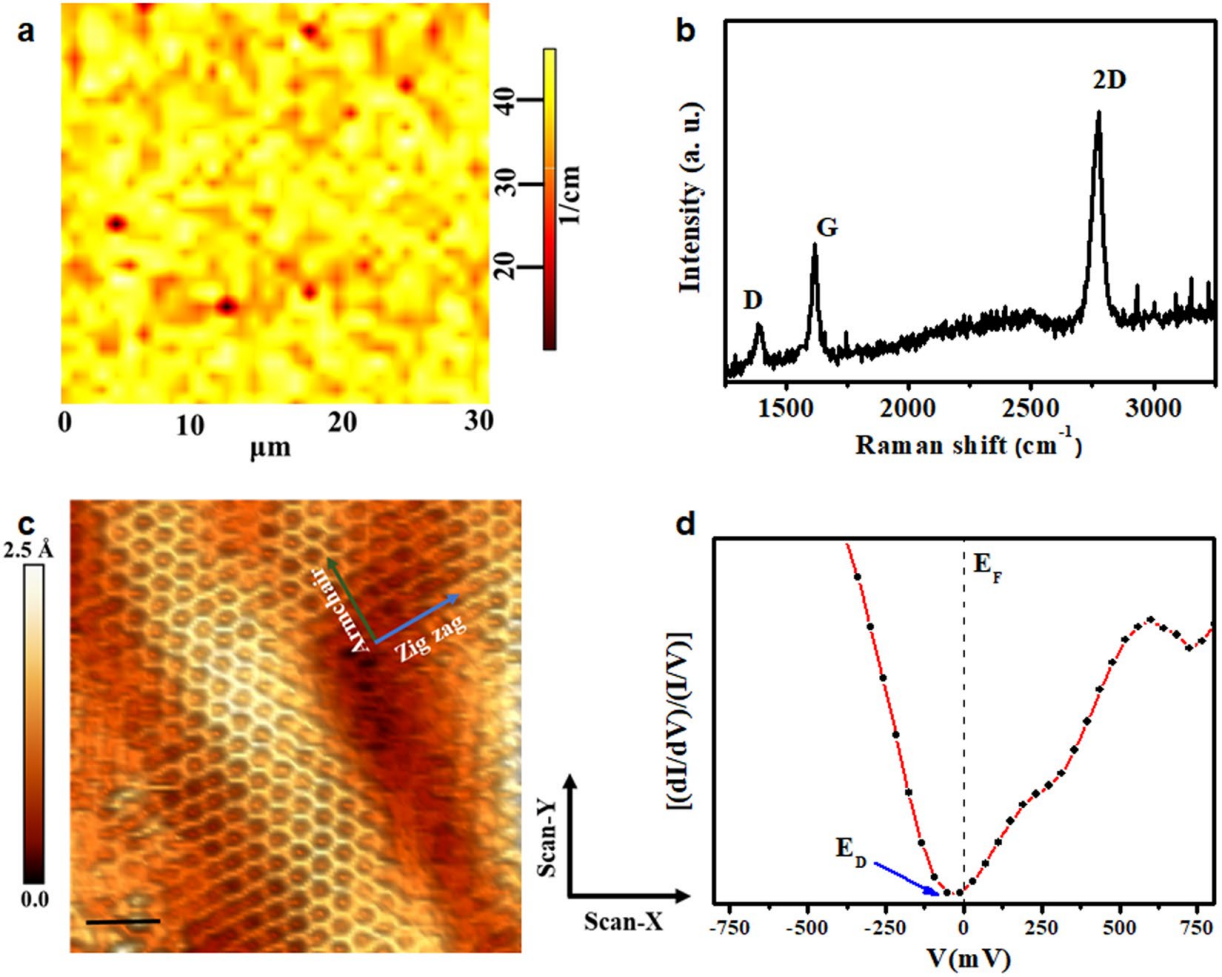
Characterization of graphene grown on Ge(110) surface. (**a**) Two-dimensional Raman map of FWHM (2D-band) over an area of 30 × 30 µm^2^ carried out on every sample; (**b**) characteristic Raman spectrum of single-layer graphene; (**c**) the honeycomb structure of graphene/Ge obtained by STM shows the sinusoidal ripples (out-of-plane deformations). The black scale bar is 1.0 nm; (**d**) STS showed as a (dI/dV)/(I/V) vs V curve. The dotted line indicates the Fermi level (E_F_) and the blue arrow indicates the position of the Dirac point E_D_. The tunneling conditions of (**c**) were V = 500 mV, I = 0.5 nA. The axes inserted in (**c**) are the specified zigzag (blue arrow) and armchair (green arrow) directions, while X- and Y- scan axes are aligned to the cleavage plane of the Ge substrate, and perpendicular to the main axis of the tip.

**Figure 2:**
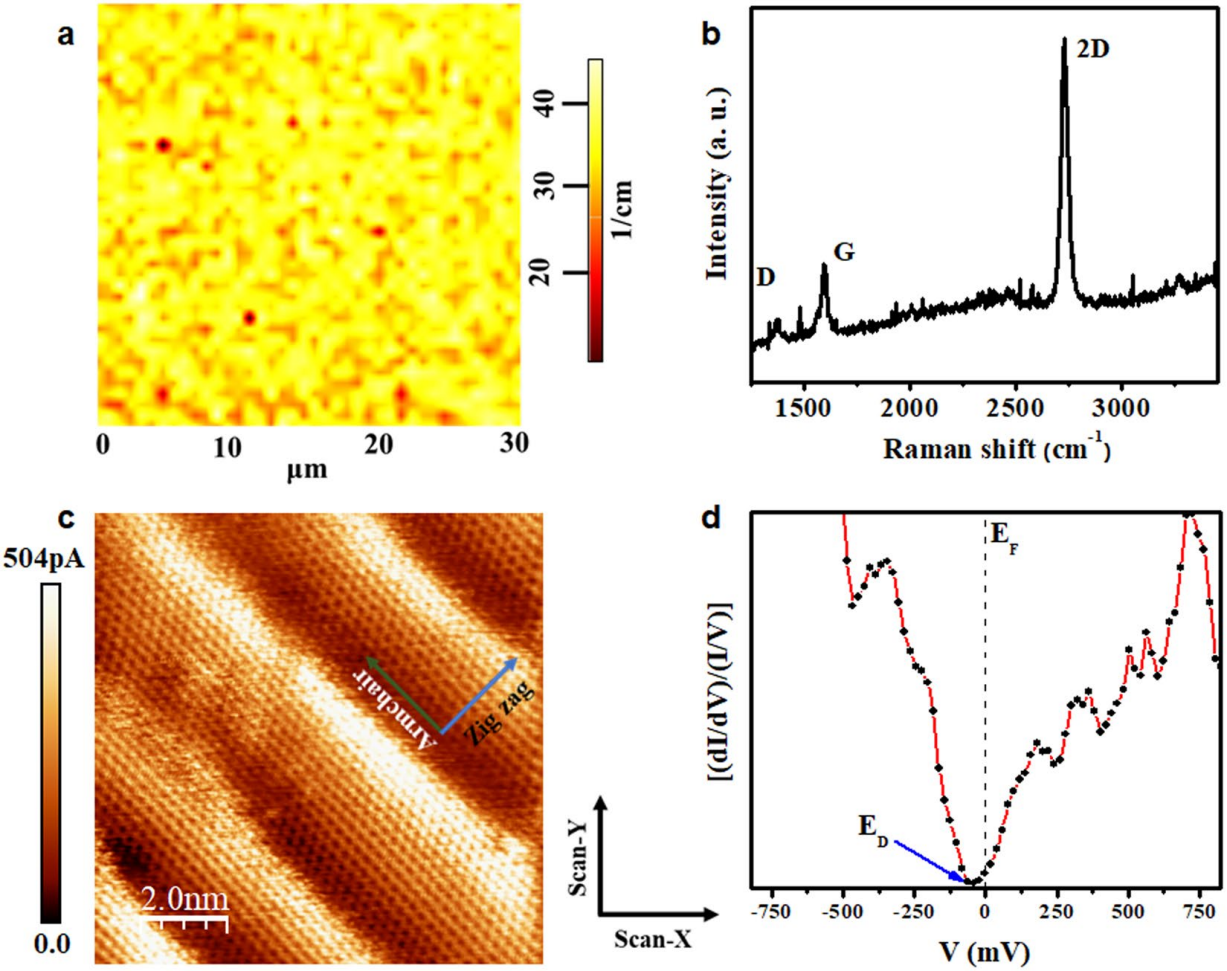
Characterization of graphene grown on Ge(100) surface. (**a**) Two-dimensional Raman map of FWHM (2D-band) over an area of 30 × 30 µm^2^ carried out on every sample; (**b**) characteristic Raman spectrum of single-layer graphene; (**c**) the honeycomb structure of graphene/Ge obtained by STM shows the sinusoidal ripples (out-of-plane deformations); (**d**) STS showed as a (dI/dV)/(I/V) vs V curve, the dotted line indicates the Fermi level (E_F_), while the blue arrow indicates the position of the Dirac point E_D_. The tunneling conditions of (**c**) were V = 25 mV, I = 0.5 nA. The axes inserted in (**c**) are the specified zigzag (blue arrow) and armchair (green arrow) directions, while X- and Y- scan axes are aligned to the cleavage plane of the Ge substrate, and perpendicular to the main axis of the tip.

The quality of the films grown on Ge was determined by Raman maps. The representative spectra obtained at several positions and randomly distributed in the samples are shown in the Fig. 1(a,b) for Ge(110) and Fig. 2(a,b) for Ge(100), respectively. These spectra exhibit typical spectral features of graphene, i.e., the 2D- (~2750 cm^−1^), G- (~1600 cm^−1^) and D- (~1350 cm^−1^) bands. In these spectra the O_2_ (~1557 cm^−1^)- and N_2_ (~2329 cm^−1^) peaks due to atmospheric oxygen and nitrogen were fitted and removed (see Fig. S2, in the Supplementary Information). These peaks were used as a standard for the calibration of the spectra.

We could determine that the graphene films were single-layer, by taking into account both the symmetry and Full Width at Half Maximum (FWHM) of the 2D-band fitted with a single Lorentzian curve with FWHM (39 ± 6) cm^−1^ for graphene grown on Ge (110) and (33 ± 4) cm^−1^ for the growth on Ge(100), and the 2D- and G-bands intensity ratio (I_2D_/I_G_ ≥ 2.0), see Figs S1(c) and S3 in Supplementary Information. On the other hand, the intensity ratio between D and G bands is used to indicate that the graphene structure has some level of disorder. In the spectra shown in Figs 1(b) and 2(b), the I_D_/I_G_ ratios are around 0.20.

The STM images shown in Figs 1(c) and 2(c) reveal the honeycomb structure of graphene and confirm the success of single-layer graphene synthesis process on germanium surfaces. Several images are made in different films (graphene/Ge(110) and graphene/Ge(100)) and all clearly demonstrate the presence of ripples (out-of-plane deformations), which are quite similar to those shown in Figs 1(c) and 2(c), respectively. In the Supplementary Information, STM images with a larger scan size are shown in the Figs S4(a) and S7. Those deformations have the same direction of propagation (wave front) as the zigzag direction and are aligned with the armchair direction independently of the randomness of its distribution on both surfaces. The axes inserted in both Figures (c) are the specified zigzag (blue arrow) and armchair (green arrow) directions, while X- and Y- scan axes are aligned to the cleavage plane of the Ge substrate, and perpendicular to the main axis of the tip.

**Figure 3:**
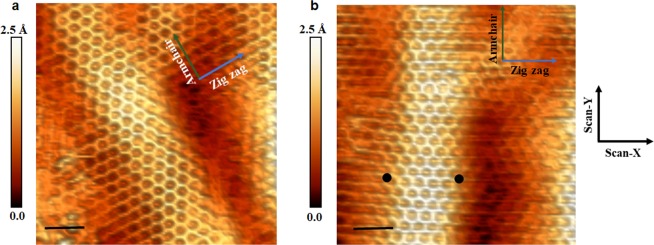
Honeycomb structure and ripples of graphene on Ge(110) obtained by STM. (**a** and **b**) Are images performed on the same area of the film. They clearly show the ripples in single-layer graphene. The image shown in (**b**) was obtained with +30° tilt in the scan direction with respect to (**a**) image. The image in (**a**) is the same shown in Fig. 1(c). The tunneling conditions for (**a** and **b**) were the same: V = 500 mV, I = 0.5 nA. The black scale bars in the figures have 1.0 nm. The black dots in (**b**) indicate the points where STS curves were taken. The axes inserted in (**a** and **b**) are the specified zigzag (blue arrow) and armchair (green arrow) directions, while X- and Y- scan axes are aligned to the cleavage plane of the Ge substrate, and perpendicular to the main axis of the tip.

**Figure 4:**
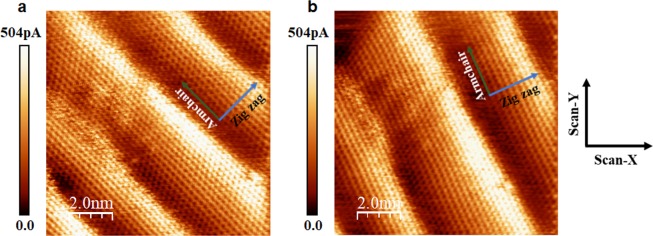
Honeycomb structure and ripples of graphene on Ge(100) obtained by STM. (**a** and **b**) Are images performed on the same area of the film. They clearly show the ripples in single-layer graphene. The image shown in (**b**) was obtained with +30° tilt in the scan direction with respect to (**a**) image. The image in (**a**) is the same shown in Fig. 2(c). The tunneling conditions for (**a** and **b**) were the same: V = 25 mV, I = 0.5 nA. The axes inserted in (**a** and **b**) are the specified zigzag (blue arrow) and armchair (green arrow) directions, while X- and Y- scan axes are aligned to the cleavage plane of the Ge substrate, and perpendicular to the main axis of the tip.

The deformations observed in the Figs 3 and 4 cannot be associated to a scan effect of the tip on the samples. We performed images on two different scan directions, 0° and 30°, in respect to the zigzag direction and at different times (see Figs 3 and 4 from (a) to (b) respectively), in order to better visualize the ripples. They showed that the ripples are independent of the scan direction and such deformations were stable during the analysis time (Supplementary Information, Fig. S6).

Local density of states (LDOS) of graphene/Ge interface was studied by STS at room temperature on every sample. Figures 1(d) and 2(d) show STS curves that are an average obtained from STS results measured at different points on the top of the corrugations shown in Figs 1(c) and 2(c), respectively. Indeed, the LDOS shows a Dirac point (E_D_) shifted around 50 meV, standard deviation of 20 meV, to the left from the Fermi level (E_F_), indicating that, at the top of the ripples, graphene films are slightly n-type doped.

**Figure 5:**
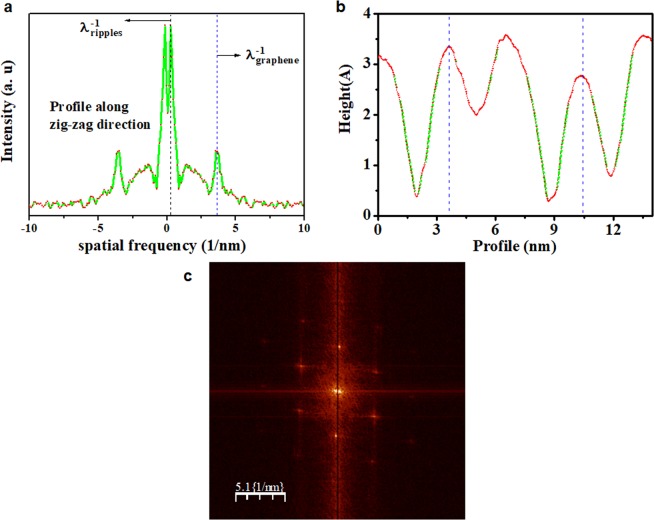
Morphology of the ripples by profile from films grown on both Ge substrates. (**a**) Characteristic profile in spatial frequency from Fig. S4(a), where λ^−1^_*graphene*_ (~3.9 nm^−1^) is associated to periodicity of graphene on Ge(110) and λ^−1^_*ripples*_ (~0.27 nm^−1^) is associated to wavelength of ripples (1D- Fast Fourier transform (FFT) was along the zig-zag direction), while the profile of ripples showed in (**b**) was determined in the graphene on Ge(100) from Fig. S4(b). (**c**) Is 2D- Fast Fourier transform (FFT) of the image shown in Fig. S4(a).

The morphology of the ripples was determined through STM images and by means of analysis of profiles of the Fig. 5(a,b). The profile in 5(a) is the one-dimensional- Fast Fourier transform (1D-FFT) from image in Figure S4(a) related with the ripples in graphene/Ge(110). It was determined along the zig-zag direction. This profile contains information about two kinds of periodicities in the image; first one is λ^−1^_*graphene*_ (~3.9 nm^−1^) which is the lattice constant of graphene and second one is λ^−1^_*ripples*_ (~0.27 nm^−1^) which is the wavenumber of the ripples. The two-dimensional-FFT of the same image is shown in Fig. 5(c), confirming the honeycomb structure of the single-layer graphene. On the other hand, the profile in 5(b) to graphene/Ge(100) from image in the Fig. S4(b) helps determining that the ripples are sinusoidal with height and wavelength of ~0.3 nm and ~3.6 nm, respectively.

**Figure 6:**
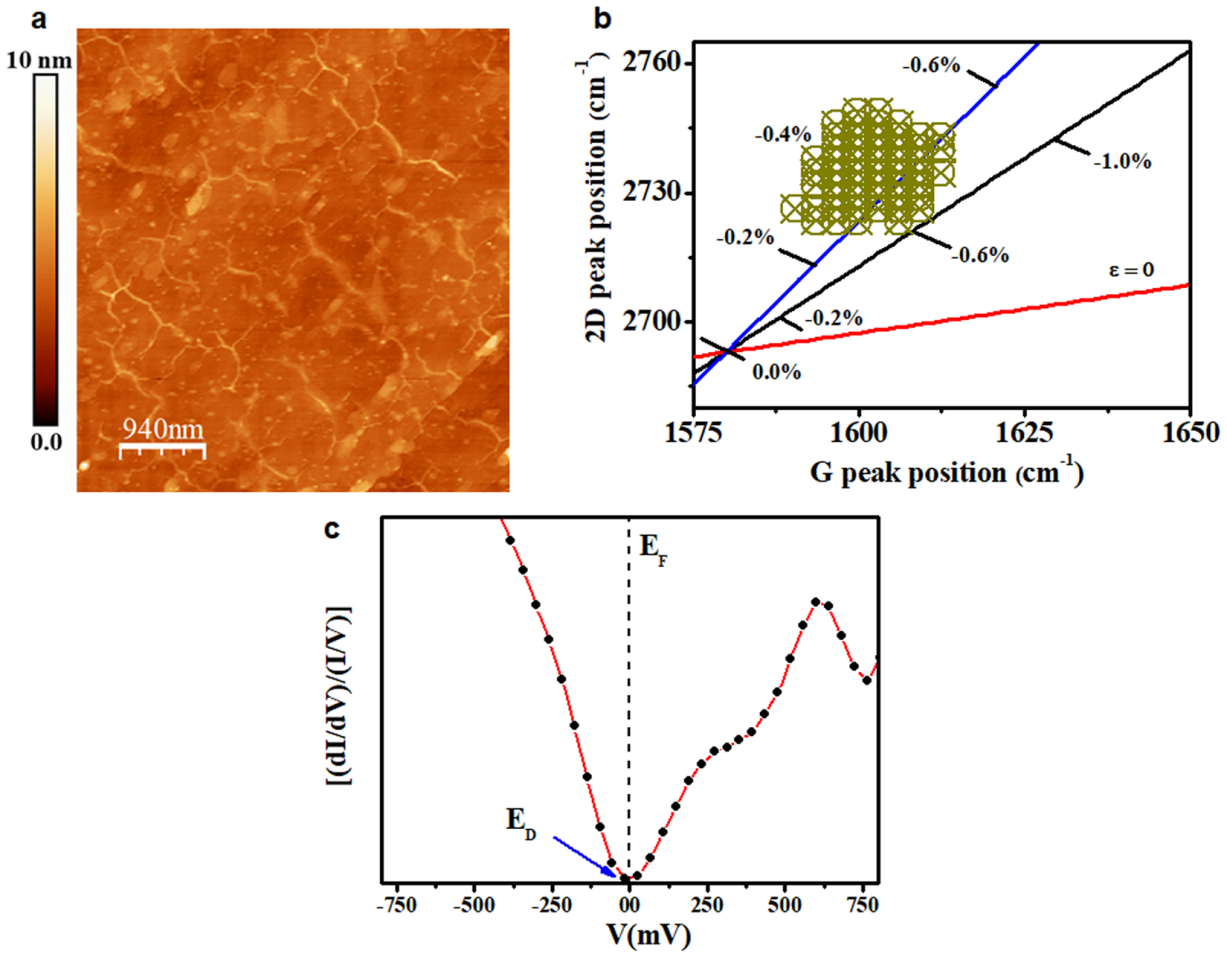
Strain-plot, topography and LDOS of graphene/Ge(110). (**a**) Topography image of sample surface made by contact mode AFM showing domain boundaries; (**b**) plot of the 2D vs G-band positions for the graphene film. Coloured lines indicate the E_2D_ and E_G_ relationship for strained undoped (biaxial-strain is represented by the blue line and uniaxial strain by the black line), and unstrained p-doped (ε = 0, red line) graphene. The neutrality point (zero point) was taken from literature^39^, and corresponds to the expected 2D and G positions for suspended freestanding single-layer graphene; (**c**) (dI/dV)/(I/V) curve measured at the black dots of Fig. 3b. The dotted line indicates the Fermi level (E_F_) and the blue arrow indicates the position of the Dirac point (E_D_).

Although the synthesis procedure was performed close to the melting point of germanium, it was possible to obtain uniform and high-quality graphene films. Figure 6(a) shows the topographic image of graphene/Ge(110) obtained by AFM. It is possible to observe the domain boundaries and the lack of facets on the surface due to the synthesis process of graphene on Ge(110), this result is in good agreement with previous works^17,18^. The roughness of this sample was determined over an area of 20 × 20 µm^2^ (*from the* Fig. S5(a) *in* Supplementary Information) and its value was of (0.8 ± 0.2) nm, i.e., the graphene/germanium interface is flat throughout the scanned area. Similar roughness was obtained from the surface of Ge (110) submitted to the same thermal cycle used for graphene growth, as can be seen in the Supplementary Information, Fig. S5(c). These results are quite different from those determined for Ge(100), since its surface is faceted during the graphene growth^18,22,23^. The formation of the Ge(107) facets was determined by different microscopy techniques and the roughness values are in the order of ~70 nm for bare Ge(100) and ~30 nm for graphene/Ge(100)^22^. On the other hand, for graphene grown on Ge(110) there is no such rearrangement (facet) of the Ge surface, so the system is quite flat.

Raman spectroscopy may be used to provide an estimate of the strain in graphene by analysing the positions of the 2D and G peaks. Figure 6(b) shows the positions of the 2D peak versus positions of the G peak taken at different points across the surface of the graphene grown on Ge(110). Following the procedure adopted in^22^, we can estimate the type of strain (uniaxial or biaxial) of the sample. The neutrality point (zero point) was taken from literature^38^, and corresponds to the expected 2D and G positions for suspended freestanding single-layer graphene. The experimental data are positioned in the region of neutrality of charge between the black and blue lines. We can say that the strain (ε) was compressive biaxial in the range between −0.2% and −0.6% for graphene but centred at −0.4%. These results were similar to those reported in^20^ for Ge (110). The origin of this compressive strain in graphene can be attributed to the opposite polarity of the thermal expansion coefficients between graphene (−8 × 10^−6^ K^−1^) and germanium (6 × 10^−6^ K^−1^) in the cooling step of the synthesis by CVD.

Knowing that out-of-plane deformations present in the graphene films can be enough to cause distortions of the atomic bonds on ripples and promote displacement in ‘π’ orbitals, we analysed the Fig. 5(a,c), which shows a small distortion in the graphene lattice. Figure 6(c) shows the LDOS at the nodes (nearly at valley) of the ripple taken at the black points from Fig. 3(b), where E_F_ and E_D_ coincide in the same energetic point (as in undoped, freestanding graphene). This result was different of what was observed in the Fig. 1(d), which was obtained upon the ripple.

Recently, theoretical calculations using a molecular dynamics approach to model the formation of ripples in graphene have shown that sinusoidal ripples appear to arise in the presence of compressive biaxial strain, as indicated by Raman results, and the presence of these ripples induces spontaneous n-doping^39^. Our STS results showed that the ripples on graphene layer are slightly n-doped, while the valleys between two of them were undoped, in good agreement with the theoretical conclusions. These results can be compared with STM/STS results obtained from CVD graphene on copper, where the authors suggested that the constraint imposed at the boundaries between the intrinsic and the n-doped regions plays a vital role in creating these 1D ripples^40^. In this case, the n-doped region is generated by the introduction of nitrogen atoms, creating a defective zone that relaxes the ripples while the undoped region remained strained and with ripples near the boundaries. Recently, it was suggested that applied strain along the zig-zag direction results in the band gap opening in graphene^32^. However, since our STS results were obtained at room temperature, in this experiment we were not able to determine the opening of the band gap if it is below 50 meV.

## Conclusion

In this work, we have performed surface characterization of single-layer graphene grown on Ge substrates with orientations (110) and (100) by CVD. STM images clearly show the presence of sinusoidal ripples with ~3.6 nm wavelength and ~0.3 nm in height, independently of the crystallographic orientation of the substrates. Those deformations have the same direction of propagation (wave front), the zigzag direction, and are aligned with the armchair direction independently of the randomness of its distribution. We suggested that these sinusoidal ripples appear to arise in the presence of compressive biaxial strain, as revealed by Raman results, due to thermal expansion coefficients with different polarities of graphene and germanium substrates.

The STS measurement results on the ripples showed a linear dispersion relation with the Dirac point, slightly shifted with respect to the Fermi energy, indicating that these out-of-plane deformations were n-doped, while the graphene regions between the highs were undoped. These periodic alternances between undoped and n-doped regions throughout the entire surface of graphene films must be taken into account in the future development of electronic devices.

## Methods

### Sample preparation

Graphene films were synthesized on undoped Ge (110) and p-doped Ge(100) substrates by CVD at semi-atmospheric pressures. The substrates were cut into small pieces (1.0 cm^2^) from 2.5 cm and 5.1 cm wafers, respectively (undoped, 30Ω.cm, single side polished supplied by Semiconductor Wafer Inc and p-type Ga-doped, 5 × 10^−3^ Ω.cm, single side polished supplied by Umicore), and cleaned with acetone, isopropyl alcohol, deionized water, respectively, and finally dried under pure N_2_ flux. After that, the Ge substrates were placed into the reactor chamber that was evacuated down to a base pressure of approximately 1.0 mPa. The growth environment was a mixture of Argon (Ar, 99.999% purity) and Hydrogen (H_2_, 99.999% purity) gases at 100 and 50 standard cubic centimetres per minute (sccm), respectively. Two sets of samples were prepared as summarized in the Supporting Information and following the step-by-step from^22^. Once the deposition temperature and pressure were reached, methane gas was introduced in the reactor, and its flux was of 1.5 and 0.5sccm for Ge(110) and Ge(100) respectively. The synthesis was carried out at 910 °C with 5 × 10^4^ Pa for both substrates, but the times were 60 and 120 minutes for Ge(110) and Ge(100), respectively. After the graphene deposition, the system was cooled to room temperature at a rate of 50 °C/s, under the same atmosphere used during the synthesis.

### Characterisation

Raman spectroscopy and AFM were performed using the integrated system from NT-MDT. Raman measurements used a micro-Raman spectrometer (NTEGRA SPECTRA), equipped with a CCD detector and a solid-state laser, which produced excitation energy of 2.62 eV. The Raman maps were taken over a 30 × 30 µm^2^ area with steps of 1.0 µm. Intensity ratios between the D, G and 2D bands were calculated using the intensity of the obtained peaks. On the other hand, AFM measurements were performed in contact mode. The topographic images were processed with a first-order plane filter while the structure in real-space and the local electronic structure of graphene were probed by STM/STS in an Omicron STM installed in an ultrahigh vacuum (~10^−8^ Pa) chamber. These measurements were simultaneously performed at room temperature using an electrochemically etched W tip.

## Supplementary information

Supplementary Information
