# Supplementary material for: CVD graphene/Ge interface: morphological and electronic characterization of ripples
Source: Sci Rep. 2019 Aug 29;9:12547. doi: 10.1038/s41598-019-48998-1 (PMC6715795; doi:10.1038/s41598-019-48998-1)
Supplement: Supplementary file 1 — Supplementary Information [file 41598_2019_48998_MOESM1_ESM.pdf]

Supplementary Information

CVD graphene/Ge interface: morphological and electronic characterization of ripples.

Cesar D. Mendoza[1]*, *Neileth S. Figueroa*[1], *Marcelo E. Maia da Costa*[1] & *Fernando L. Freire Jr*[1].

[1]Departamento de Física, Pontifícia Universidade Católica do Rio de Janeiro, 22451-900, Rio de Janeiro, RJ, Brazil.

*Corresponding author. Tel: +55 21 35271272. Fax: +55-21 35271271.

E-mail: cesar.diaz@vdg.fis.puc-rio.br (Cesar Diaz)

Experimental details:

In the manuscript, we report data related to the optimal conditions of graphene synthesis on Ge(110) and Ge(100) respectively. For the case of Ge(110): the CH_4:H_2 flow ratio and time were 0.03 and 60 minutes, respectively. To determine the best synthesis condition, we investigated the influence of time and CH_4:H_2 flow ratio during the growth. To do this, we synthesized samples at different times ranging from 15 to 90 minutes, maintaining the CH_4:H_2 flow ratio at 0.03 and the temperature at 910°C. Raman spectra taken from samples grown at 30 minutes and 90 minutes were identical, see Figure S1(a). This implies that the graphene growth on Ge(110) in our setup is self-limiting and is a surface-mediated process instead of a precipitation process.

After setting the growth time at 60 minutes and the temperature at 910°C, we varied the CH_4:H_2 flow ratio from 0.30 to 0.03, as shown in figure S1(b). One can see that the D-band intensity and the FWHM (Full Width at Half Maximum) of the 2D-band decrease as a function of the decreasing in the CH_4:H_2 flow ratio, see Figure S1(c) and (d). We did not observe graphene growth for flux of 0.01 CH_4:H_2 on Ge(110). On the other hand, the graphene growth on Ge(100) was carried out as in reference [22] of the manuscript: the CH_4:H_2 flow ratio, growth time and temperature were 0.01, 120 minutes and 910°C, respectively.

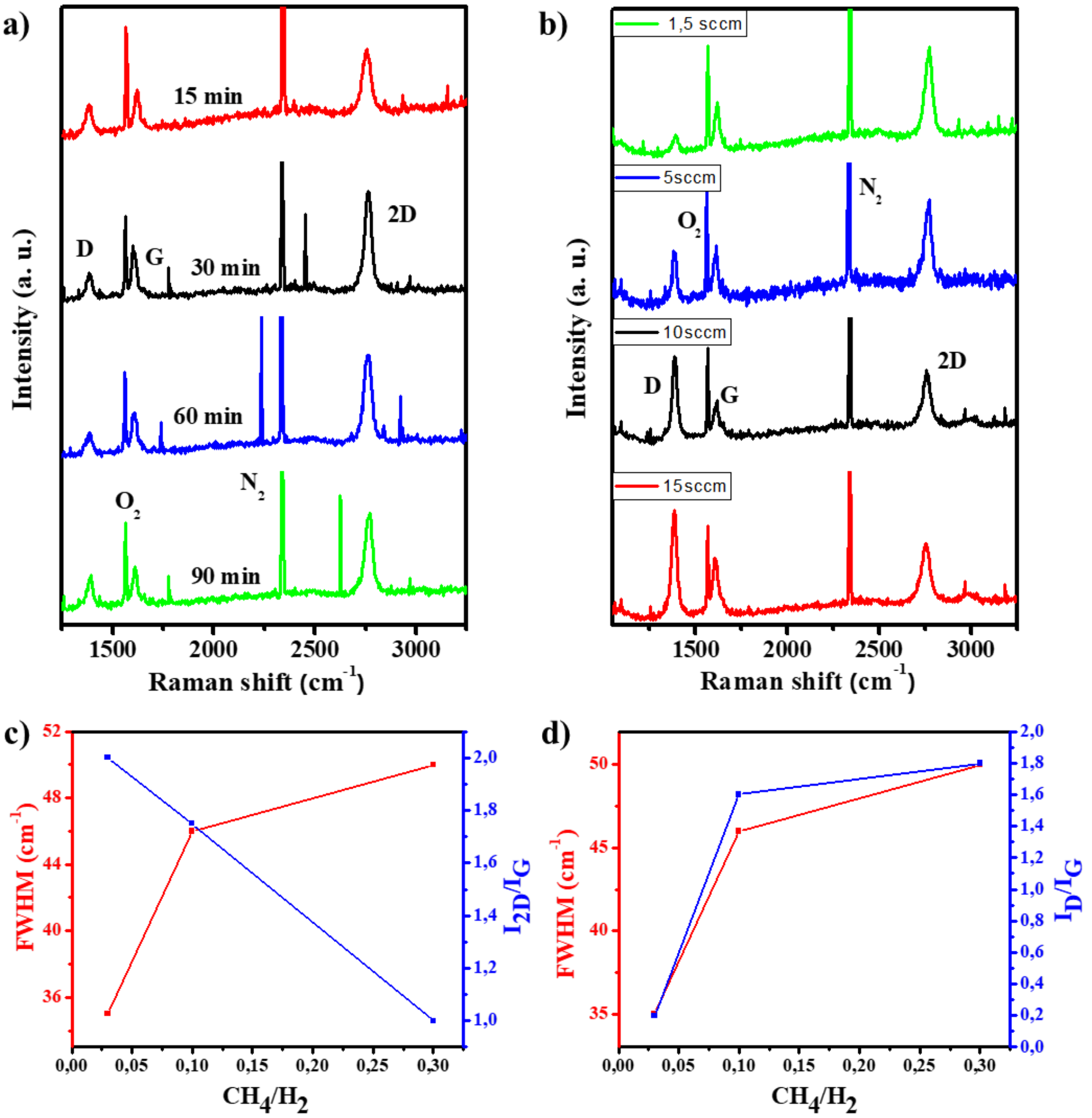

Figure S1: Raman spectra in (a) are graphene samples grown on Ge(110) using H_2:CH_4 flow ratio of 0.03 and temperature of 910°C. Growth times were 15 minutes (red line), 30 minutes (black line), 60 minutes (blue line) and 90 minutes (green line). In (b) Raman spectra of graphene synthesis on Ge(110) using different CH_4:H_2 flow ratios. (c) FWHM of the 2D-band and the intensity ratio of the 2D and G bands as a function of the CH_4:H_2 flow ratios. (d) FWHM of the 2D-band and the intensity ratio of the D and G bands as a function of the CH_4:H_2 flow ratios. For (b), (c) and (d) the growth temperature and time were 910ºC and 60 minutes, respectively.

Figure S2 shows the spectra before and after the fit and removal of O_2 (~1557 cm^{-1})- and N_2 (~2329 cm^{-1})-peaks associated with atmospheric oxygen and nitrogen.

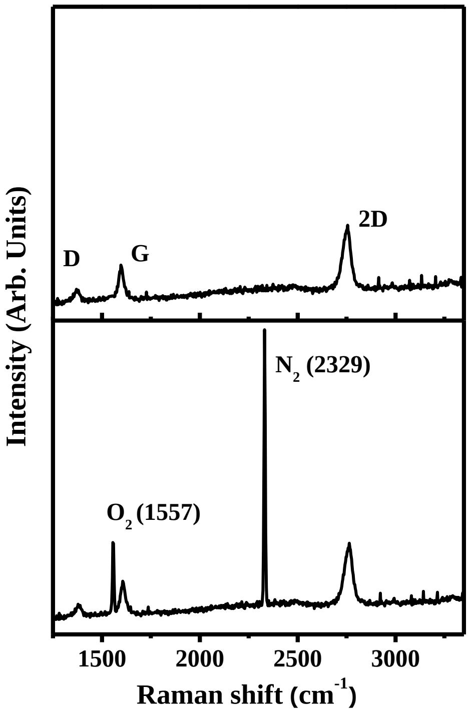

Figure S2: Raman spectra of graphene/Ge(110) before fitting and removing the O_2 (~1557 cm^{-1})- and N_2 (~2329 cm^{-1})-peaks associated with atmospheric oxygen and nitrogen (bottom spectrum), and after their removal (top spectrum).

Figure S3 presents the FWHM (2D-band) distribution of the Raman maps shown in Figure 1(a) and 2(a) respectively, while Figure S4 shows the images from where profiles seen in Figure 5 of the manuscript were taken.

Figure S5 shows the topographic images for graphene/Ge(110) sample in an area of 20 x 20 µm^2, (b) is the image of Ge(110) sample after having been heated to 910ºC for 60 minutes in the same growth environment (but without CH_4) and the image (c) is the same area as that shown in the Figure 1(c) of the manuscript.

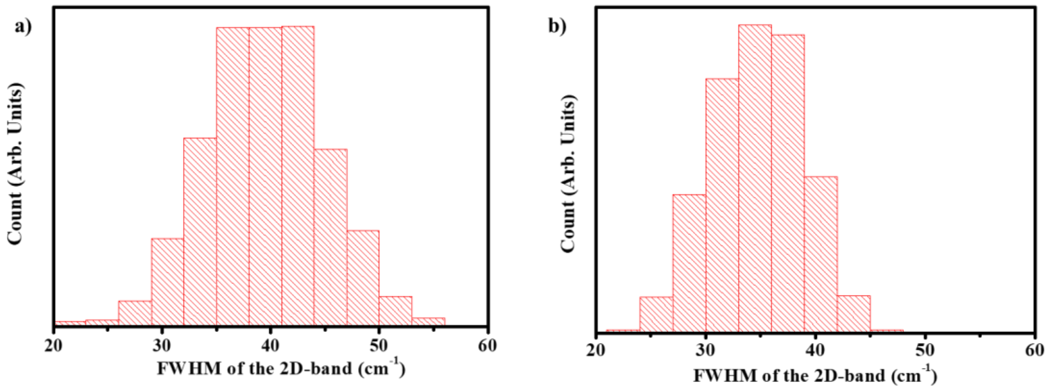

Figure S3: FWHM (2D-band) Distribution of the Raman maps of graphene shown in Figure 1(a) and 2(a) for both Ge orientations.

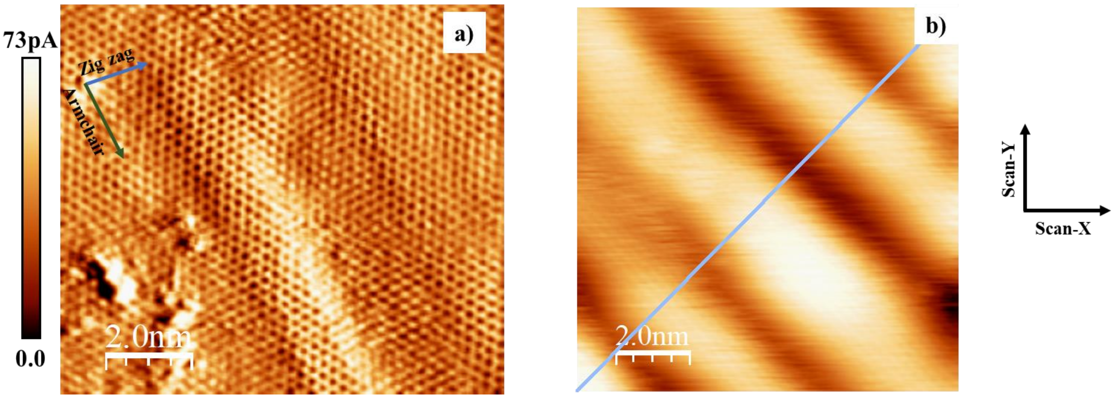

Figure S4: (a) is the image of graphene/Ge(110) from which was taken the characteristic profile in frequency shown in Figure 5(a), while (b) is the height image of the graphene on Ge(100) to determine the profile of ripples shown in the Figure 5(b). The tunnelling conditions for (a) and (b) were V = 150mV, I = 0.75nA and V = 25mV, I = 0.5nA. The axes inserted in (a)-(b) are the specified zigzag (blue arrow) and armchair (green arrow) directions, while X- and Y- scan axes are aligned to the cleavage plane of the Ge substrate and perpendicular to the main axis of the tip.

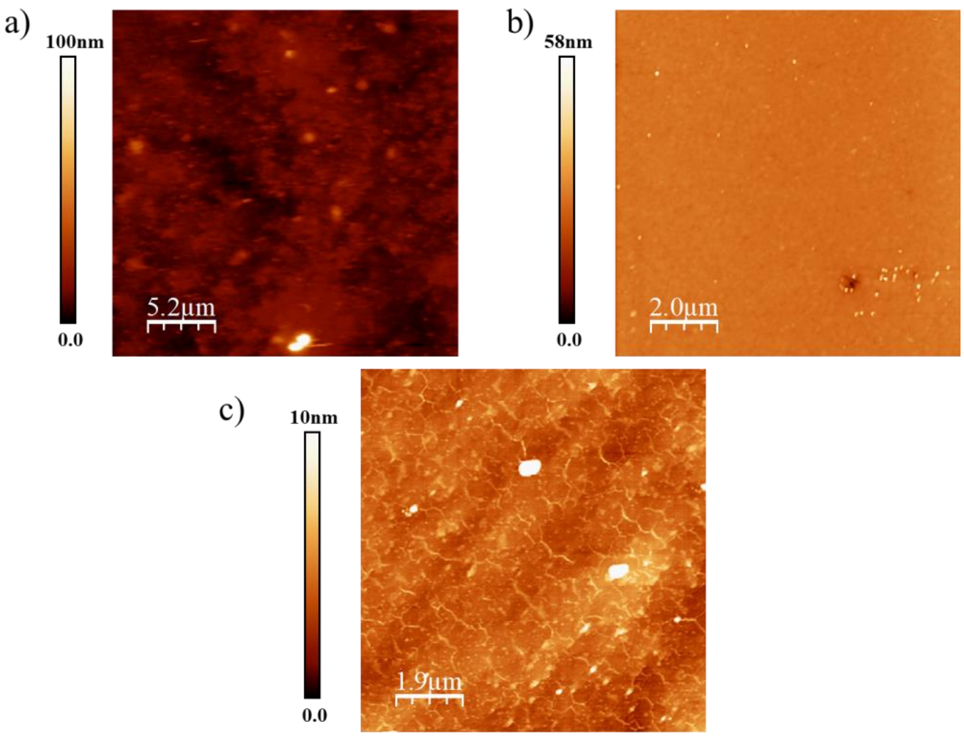

Figure S5: Topographic images by AFM: (a) graphene/Ge(110) sample in an area of 20 x 20 μm^2 with roughness of 0.8 ± 0.2 nm. (b) Ge (110) sample, after heating up to 910ºC for 60 minutes in the same growth environment (but without CH_4) with roughness of 1.8 ± 0.3 nm and (c) shows the same area that appears in the Figure 1(c), but in a larger size, its roughness was of 0.6 ± 0.2 nm.

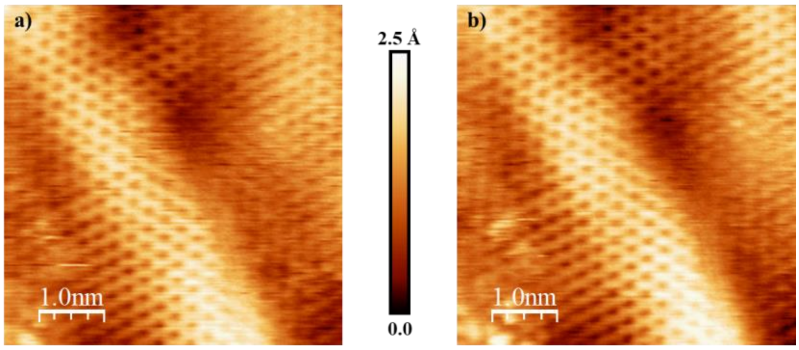

Figure S6: (a) and (b) are images performed on the same area of the film at different times. They clearly show the ripples in single-layer graphene. The image shown in (b) was obtained with the difference of 60 minutes with respect to (a). The image in (a) is the same shown in Figure 1(c). The tunnelling conditions for (a) and (b) were the same: V = 500mV, I = 0.5nA.

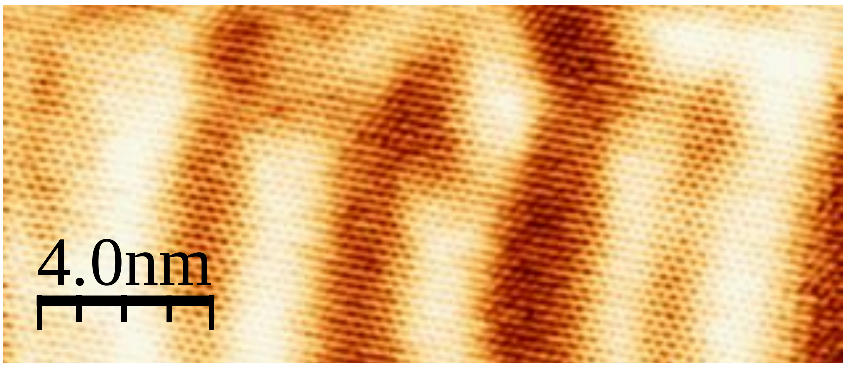

Figure S7: Image performed on graphene/Ge(100) with a scan-size of 20x10nm^2. It clearly shows the ripples in single-layer graphene. The tunneling condition was V = -25mV, I = 1.5nA.
